# Nanoporous Silica-Based Protocells at Multiple Scales for Designs of Life and Nanomedicine

**DOI:** 10.3390/life5010214

**Published:** 2015-01-19

**Authors:** Jie Sun, Eric Jakobsson, Yingxiao Wang, C. Jeffrey Brinker

**Affiliations:** 1 Beckman Institute for Advanced Science and Technology, University of Illinois at Urbana-Champaign, Urbana, IL 61801, USA; E-Mails: jiesun2@illinois.edu (J.S.); jake@illinois.edu (E.J.); 2 Department of Bioengineering, University of California San Diego, La Jolla, CA 92093, USA; E-Mail: yiw015@ucsd.edu; 3 Department of Chemical and Nuclear Engineering, Molecular Genetics and Microbiology, University of New Mexico, Albuquerque, NW 87106, USA; 4 Sandia National Laboratories (SNL), Albuquerque, NW 87185, USA

**Keywords:** protocell, nanoporous silica, synthetic biology, supported lipid bilayer, FRET, nanomedicine, designs of life

## Abstract

Various protocell models have been constructed *de novo* with the bottom-up approach. Here we describe a silica-based protocell composed of a nanoporous amorphous silica core encapsulated within a lipid bilayer built by self-assembly that provides for independent definition of cell interior and the surface membrane. In this review, we will first describe the essential features of this architecture and then summarize the current development of silica-based protocells at both micro- and nanoscale with diverse functionalities. As the structure of the silica is relatively static, silica-core protocells do not have the ability to change shape, but their interior structure provides a highly crowded and, in some cases, authentic scaffold upon which biomolecular components and systems could be reconstituted. In basic research, the larger protocells based on precise silica replicas of cells could be developed into geometrically realistic bioreactor platforms to enable cellular functions like coupled biochemical reactions, while in translational research smaller protocells based on mesoporous silica nanoparticles are being developed for targeted nanomedicine. Ultimately we see two different motivations for protocell research and development: (1) to emulate life in order to understand it; and (2) to use biomimicry to engineer desired cellular interactions.

## 1. Introduction

Synthetic biology is an emerging field with the overall goal to understand life processes using an engineering approach [1]. A powerful recent approach to synthetic biology has been the design, synthesis, and assembly of a complete genome and its transplantation into a recipient cell to create a new line of engineered cells [2]. While the genomes are known and can be built from scratch in this top-down approach, the myriad regulatory, metabolic, and signaling networks are not completely characterized. These top-down approaches have limitations for the understanding of fundamental molecular regulation, since the host organisms have a complex and incompletely defined molecular composition. Alternative biochemical methods using cell-free extracts or purified components are too simple to mimic cellular environments. Thus, understanding the detailed mechanism of the top-down engineered cell suffers from the existence of uncontrolled variables. 

A complementary approach for the engineering of living cells is the bottom-up synthesis of a cell from defined building blocks that may be as small as individual genes, proteins, and RNAs, utilizing a “protocell” platform built by directed self-assembly that provides for independent definition of the cell interior and the surface membrane [3,4]. We focus our review on a complementary bottom-up approach to engineer protocells using biomaterials or synthetic materials to mimic the living cells, with each component of the system precisely defined and controlled. This approach will allow us to work at the levels of both protein and gene regulation in a cell-like environment. This bottom-up approach is precisely complementary to the top-down approach. Although the composition of a bottom-up cell would be significantly simplified compared to a living cell, the bottom-up cell can be precisely controlled with adjustable variables. This single cell entity can serve as a multi-scale machine capable of perceiving external environmental cues and guiding the regulation of signaling transduction and gene/protein production inside the cells. A computational model can be established to provide the molecular insights by which the interactions between different components of the cell entity are programmed. We envisioned that the bottom-up and top-down approaches will ultimately converge on a minimal living cell, whose operation will be generally unraveled during the processes of constructing simple building blocks and bottom-up cell entities. 

This bottom-up approach is not entirely new as many other protocell designs have been used to understand life, such as liposomes, polymersomes, inorganic and hybrid protocells. Liposomes are the first and most widely used protocell design as the major components of the membranes of living cells are lipids [5]. In addition, the enclosed nominally spherical structure of liposomes provides basic compartmentalization and definition of interior and exterior. Two categories of liposomes have been made, the ones made of fatty acids or phospholipids, for different purposes. Fatty-acids based liposomes are postulated to be good models for prebiotic precursors to modern cells and may therefore shed light on the origin of life [6,7]. As the main building blocks of modern cells’ membranes are phospholipids, liposomes made of synthetic or natural phospholipids have been the major protocell design for both reconstitution of membrane proteins, biochemical reactions and supramolecular assembly inside the liposomes [8,9,10,11,12]. Even though lipids are the most biomimetic choice for protocells, an emerging area of interest is discovering new membranes made of self-assembling polymers or proteins [13,14,15]. Consideration of these molecules not only greatly expands the library of molecules for membrane assembly, but also expands the repertoire of possible membrane properties, since other polymers exhibit some properties not possible with lipids. These polymersomes have been shown to be practical micro- or nano-reactors, accommodating even protein synthesis inside [16]. Other than organic materials for membranes, inorganic materials were also explored as a membrane material [17,18]. Silica nanoparticles coated by copolymers can electrostatically gate membrane permeability [19]. Importantly, hybrid protocells combining different compositions can be constructed. Liposomes with internal and fatty acid-coated coacervate microdroplets are examples of hybrid protocells [20,21]. Details on different protocell designs and their applications have been reviewed elsewhere [5,13,17]. The remainder of this review focuses on one type of hybrid protocell: nanoporous silica encapsulated within a supported lipid bilayer.

## 2. Design, Synthesis and Advantages

This class of protocells is comprised of rudimentary cell-like constructs formed by liposome fusion on a porous spherical silica particle [3] or a silica replica of a cell [22]. They have been recently shown in cell culture to be a useful tool for targeted drug delivery [4]. Compared to giant unilamellar vesicles (GUVs), which have been studied extensively as cell mimics [23,24,25,26], they offer several advantages: (1) As with natural cellular plasma membranes, the membrane bilayer of the protocell is supported on a porous, cytoskeleton-like scaffold. The membrane-scaffold interaction confers enhanced stability, lateral diffusivity and fusogenicity; (2) Proteins and other membrane-bound components can be more readily incorporated into the supported lipid bilayer (SLB) than into comparable GUV’s to achieve membrane-like functionality; (3) Protocells can be easily loaded with nucleic acids, proteins, and messenger molecules to mimic the cytoplasm by relatively simple immersion procedures. Association of biomolecular components with the high surface area of internal scaffold allows for high loading and mimicking of the crowded environment of living cells, known to impact intracellular reactions [27,28,29], while maintaining accessibility and diffusivity of ions and molecules; (4) The particle surface and internal scaffold charges as well as hydrophobicity can be independently varied via well-controlled silane coupling chemistry utilizing organosilane and bridged silsesquioxane silica precursors; (5) Through tailored electrostatic interactions, the SLB asymmetry and the orientation of transmembrane proteins can be controlled; (6) The “intracellular” pKa and the extent of hydrogen bonding and electrostatic interactions with biomolecular cargos can be well controlled [30,31,32]. The combination of these features, derived from the porous scaffolds and SLB, distinguishes nanoporous silica-based protocells from “hollow” liposomes or vesicles and membrane-free nanoparticles or droplets as a promising alternative platform for synthetic biology. Furthermore, like a cell, the nanostructured silica particle is optically transparent, allowing cutting edge fluorescence imaging techniques, such as fluorescence resonance energy transfer (FRET), to be readily applied to study real-time molecular functions within protocells.

The protocell fabrication process consists of synthesis of the silica particle core according to a myriad of “sol-gel” and self-assembly approaches developed in our laboratory, followed by loading of biomolecular cargos and liposome fusion to form a supported lipid bilayer surrounding the core. The cores are formed principally by an aerosol-assisted self-assembly process [33], wherein aerosol droplets are generated, transferred under laminar flow through drying and heating zones, and collected. Starting with an initially homogeneous ethanol-water solution of soluble silica plus amphiphilic surfactants or block copolymer templating agents, with initial surfactant concentration c_0_ less than the critical micelle concentration, solvent evaporation drives radially-directed silica surfactant self-assembly into ordered periodic mesophases confined to spherical droplets [34]. Solidification of the droplet by rapid heating followed by extraction of the surfactant or block copolymer results in a porous silica replica of the mesophase characterized by an ordered periodically arranged pores that are uniform in size [33,34]. The pore size can be adjusted from 2 to over 20 nm. Particle size is controlled principally by concentration of the precursor solution, and is adjusted in order to mimic the dimension of natural cells and enable optical microscopy and spectroscopy.

**Figure 1:**
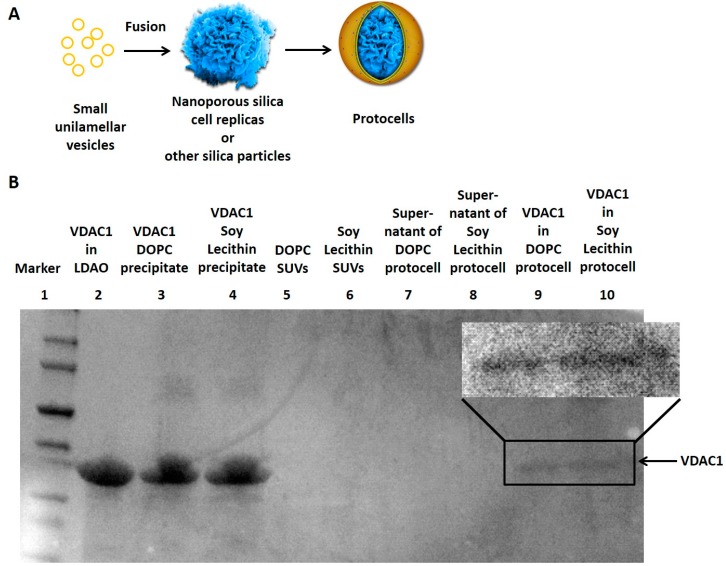
(**A**) Fusion of small unilamellar vesicles on nanoporous silica cell replicas and other silica particles forms protocells; (**B**) The sodium dodecyl sulfate polyacrylamide gel electrophoresis (SDS-PAGE) showed the VDAC1 protein (arrow) from different samples. VDAC1 successfully incorporated into the DOPC or Soy Lecithin-coated protocells (Lane 9 and 10) while the supernatant of the centrifuged protocells contained no VDAC1 (Lane 7 and 8). Most VDAC1 proteins precipitated when detergent Lauryldimethylamine-oxide (LDAO) was removed from the lipid-detergent-protein mixture (Lane 3 and 4). Original SUVs before protein incorporation had no VDAC1 (Lane 5 and 6).

Membranes are put onto protocells by incubation with liposomes (Figure 1A). Hence any chemical composition of membrane that can be formed into liposomes can be used to coat a protocell. In addition, proteins and other membrane molecules can be incorporated into the protocell membrane either before or after the liposome incubation step. The protocells, which consist essentially of the liposomes supported by nanoporous silica scaffolds, are much more robust and long-lived than ordinary liposomes [35]. In our view, protocells should be seriously considered as a general alternative to liposomes.

The newly developed cell/silica composites (CSCs) in our group have much greater fidelity to the structure of living cells than completely synthetic nanoporous silica spheres [22]. They utilize mammalian cells as the template to fabricate silica replicas capturing the remarkable 3D intra- and extracellular features of real cells with stabilized nanomaterials. These replicas are essentially indistinguishable from living cells in electron microscopy images. The replicas contain pores and cavities of a wide range of sizes in the nanometer range and the surface recapitulates the 3D nature of a natural cytoskeleton. They can be loaded with different chemicals and coated with a lipid bilayer, and therefore can be utilized as a scaffold for protocells (Figure 1A).

## 3. Nanoporous Silica-Based Protocells

### 3.1. Reconstitution of Membrane Proteins in the Lipid Bilayer of Protocells

Compartmentalization is a critical feature of living cell. It is achieved by a membrane that separates the interior of the cell from its environment with controlled exchange of ions, nutrition/sugar and energy/ATP, small molecules, and macromolecules. The selective exchange (uptake and release) of ions and materials across membranes is controlled by membrane proteins with diverse functions, such as passive transport, facilitated diffusion and active transport. Therefore, reconstitution of membrane proteins in the protocell platform (1) enables the functional study of the protein of interest in a well-defined system; (2) builds a biomimetic microreactor for controlled synthesis of inorganic multicomponent materials; (3) creates membranes with desired function of material exchange, which is essential for the design of life. Simple compartmentalized modules with partial cellular functions may also give us insight into early or minimal life forms.

Lipid vesicles have been widely used to study membrane proteins. Even though the lipid bilayers in the vesicles function as barriers, as mentioned above, they suffer from inherent instability. Supported lipid bilayers on planar or particulate solid substrates, such as silicon dioxide/silica, show enhanced stability [36,37,38]. There are several monolayers of water between the silica and the lipid bilayer, allowing the free diffusion of incorporated membrane proteins [38,39]. We hypothesize that the non-bulk water structure between the bilayer and the support accounts for one of the distinctive properties of bilayers on a nanoporous substrate; that is, increased lateral mobility of membrane molecules [40].

Several groups have pioneered the functionalization of protocell membranes. Brozik* et al.* characterized in detail the properties of lipid bilayers on an array of nanoporous silica microspheres (10–30 µm sphere size, 10–100 nm pore size) [35]. They discovered that the larger sphere size and smaller pore size correspond to increased bilayer stability. The light-driven proton pump in Archaea, bacteriorhodopsin, was successfully reconstituted in the lipid bilayer of protocells with 70% displaying the correct orientation. Functional assay with a loaded pH-sensitive dye confirmed that bacteriorhodopsin pumps proton upon photon absorption. They also tested two methods of protein functionalization on protocells: (1) proteoliposome deposition on silica surfaces; and (2) protein direct incorporation into preformed detergent-saturated supported bilayers. A serotonin receptor, 5HT3R, was purified from human embryonic kidney cells and incorporated in the protocells with each method. The second approach resulted in 94% correctly oriented 5HT3R *vs.* 74% with the first method, suggesting that interaction with the core of the protocells may help position the membrane proteins in the right orientation--another advantage of the protocell platform. One hypothesis for the effect is that the adhesion between supported lipid bilayer and nanoporous silica core may prevent large extracellular portion of the receptor from crossing the membrane. We anticipate that further improvement of precision orientation of membrane proteins may be possible by adjusting charge on the silica core. Serotonin-induced calcium release was observed in the protocells, confirming the presence of correctly oriented functional 5HT3R in protocells. Unfortunately patch clamp experiments were not possible with silica-based protocells as the rigid silica support doesn’t allow the formation of gigaseals for single-channel and whole-cell patch clamp.

Another multisubunit transmembrane functional proton pump, cytochrome c oxidase (CytocO) was reconstituted on porous silica particles (550 nm sphere size, 3 nm pore size) by Bergstrom and Brzenzinksi groups [41]. Their results showed that the protein-lipid ratio was the same in the vesicles and protocells. CytocO can be reduced selectively and the amount of reduced CytocO can be measured, providing a simple way to determine its orientation. First they used a membrane impermeable reducing agent to reduce all CytocO proteins facing outward and then added a permeable reducing agent to reduce those facing inside and then calculated the ratio of these two populations. They showed that the orientation of CytocO in vesicles is 71% ± 4%, not significantly different from 68% ± 5% in protocells. This result strongly suggests that membrane protein orientation is preserved during the fusion of liposomes with the protocell core. The formation of a transmembrane electrochemical proton gradient in CytocO-containing protocells not only confirmed the activity of the complex but also the integrity of the lipid bilayer for being impermeable to protons. Our summary inferences from the above-cited two papers are: (1) Orientation of membrane proteins in protocells is preserved during liposome fusion to the core, which may shed some light on the fusion mechanism [41]; (2) The orientation of the proteins in liposomes is not completely random (for all three proteins the reported orientation was about 70% in the biologically correct direction) [35,41]; (3) If the protein is inserted into the protocell membrane after fusion with the silica core, the orientation may be enhanced to have even greater fidelity to the biological orientation [35].

The Bergstrom group extended their work from proton transport to sodium transport and studied two membrane proteins through liposomal fusion of small unilamellar vesicles onto the nanoporous silica spheres (6 µm sphere size, 2.6 nm pore size) [42]. The large size of the silica spheres in this study allows confocal imaging to assay real-time membrane protein activities of protocells, similar to imaging live cells. Using Sodium Green, a sodium-sensitive dye that increases in fluorescence upon interaction with sodium, imaging provides spatial and temporal resolution of the sodium concentrations. The stability, durability and permeability of supported lipid bilayers with various components were tested with dyes, confirming the integrity of membranes. Passive sodium uptake and release along the chemical concentration gradient were imaged with the integration of gramicidin A (a univalent cation channel-forming peptide) in the protocell membrane. In addition, active transport of sodium by a primary sodium ion transporter ATP synthase in the presence of ATP was demonstrated using dye-loaded particles imaged using time lapse confocal laser scanning microscopy. Imaging individual protocells also showed heterogeneity of the intensity and dynamics of fluorescence inside the core, which may result from the heterogeneous concentrations of the bilayer-bound membrane proteins and variations in the pore volume and structure of the core. The heterogeneity of liposome-based protocells was discussed previously [43]. This level of resolution is not possible with protocells of nanometer size, which cannot be imaged individually due to the limit of diffraction. This study also illustrated the advantage of the porous silica spheres as support for lipid bilayer compared to solid ones, which are unable to incorporate dyes.

As ATP is an energy source for many biochemical reactions, ATP permeability of the membrane can be controlled by membrane proteins. The pore-forming toxin α-hemolysin has been shown to be permeable to ATP in liposomes [44]. Other naturally-occurring membrane proteins which are permeable to ATP have not yet been reported in protocells. The voltage-dependent anion channel 1(VDAC1), a membrane protein in the outer mitochondrial membrane, has been shown permeable to ATP [45]. Our group has found that VDAC1 can be successfully incorporated into protocell membranes composed of either DOPC, or of soy lecithin (Figure 1B). In detail, purified VDAC1 proteins in micelles [46,47] were incubated with small unilamellar vesicles (SUVs) made of either DOPC or soy lecithin first and the detergent was subsequently removed to make the VDAC1 proteoliposome. The proteoliposome was incubated with nanoporous silica particles to create protocells with VDAC1 incorporated in the membrane. The functionality of VDAC1 will be further studied in the protocell platform.

The above examples have demonstrated the protocell as a versatile platform to reconstitute membrane proteins for both functional studies and biomimetic synthesis. Up to now only single species of membrane proteins have been studied at one time in protocells even though multiple membrane proteins have been reconstituted in liposomes [48]. In the future multiple membrane proteins with related or diverse functions will likely be combined together into one protocell, mimicking the diversity and complexity of biological membranes in living cells. Additionally for membranes supported on silica cell replicas, we expect that, due to the local variation in curvature imposed by the cytoskeleton-like scaffold, lateral forces mimicking those of living systems will be generated leading to new phase behavior and life-like complexity.

### 3.2. Reconstitution of Biochemical Reactions inside Porous Silica Core of Protocells

We have discussed the reconstitution of biological membranes in protocells. The first step to reconstitute the cytosol of living cells is also being pursued. The cytosol of living cells contains thousands of soluble proteins, which carry out myriads of biochemical reactions. Liposomes have been used as a cytosol mimicking bioreactor for complicated biochemical reactions, such as polymerase chain reaction and protein synthesis [9,44]. However, the aqueous interior of liposomes cannot mimic the macromolecular crowding in living cells where biochemical reactions happen. Our group has pioneered the aerosol-assisted assembly of porous silica with 2D hexagonally ordered cylindrical channels/pores [33], which is a rough mimic of cellular cytoskeleton. Equally interestingly, we have recently developed the technology to make silica replicas of mammalian cells that preserve the detailed structural features of the living cell, including the crowded and tortuous intracellular pathways for diffusion [22]. The porous silica cell replica is a compelling alternative as the cell-templated porosity is a closer mimic of cellular cytoskeleton and simulates the effect of* in vivo* macromolecular crowding. Even though few biochemical reactions have so far been reconstituted in the lipid-bilayer-covered porous silica, various functional enzymes have been encapsulated inside porous silica, suggesting the plausibility of this idea [49].

A model system based on the protocell can allow an extracellular signal to be perceived by a membrane-bound receptor, which in turn triggers intracellular signaling cascades. This arrangement constitutes the core module of how cells respond to environmental signals. The exemplary system is the cell’s response to platelet-derived growth factor (PDGF), which regulates cell growth and division. PDGFs bind to PDGF receptors on the plasma membrane and induce dimerization of the receptors [50], which in turn modulate the activity of downstream effectors, such as tyrosine phosphatases Shp2, an important molecule associated with Noonan syndrome, LEOPARD syndrome and Chronic Myelomonocytic Leukemia [51,52,53].

**Figure 2:**
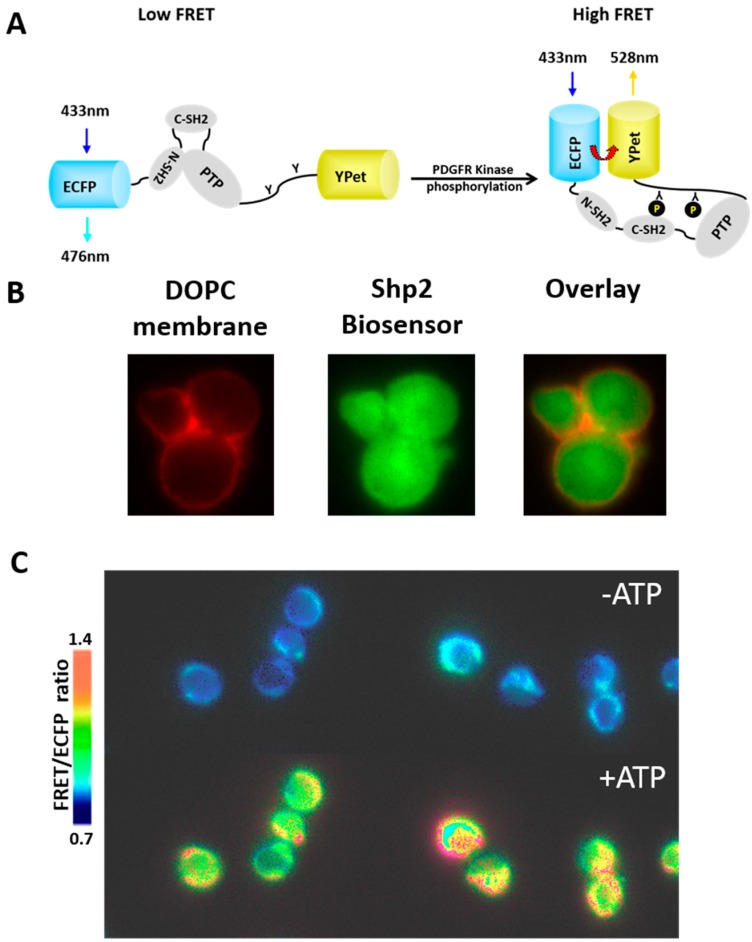
(**A**) The design of the FRET-based Shp2 biosensor; (**B**) The protocells visualized with coated DOPC membranes with 1% Texas Red- DHPE and loaded fluorescent Shp2 biosensor inside the silica cell replicas; (**C**) In the presence of loaded PDGFR kinase, the FRET of the Shp2 biosensor inside silica cells increased upon ATP addition.

Using an Shp2 biosensor based on FRET, we have found that the nature of intramolecular interactions between domains within Shp2 upon extracellular stimulation of cells by PDGF is quite different from the corresponding interaction in solution as stimulated by PDGFRβ kinase (the putative intracellular signaling domain activated by PDGF binding) [54]. The engineered cellular entity with controllable environment and compositions based on the protocell allows us to gain insights into the fundamental principles governing the molecular regulation under different environments. A FRET biosensor containing a full length Shp2 flanked by an enhanced cyan fluorescent protein (ECFP) and a yellow fluorescent protein variant (YPet) was constructed [54]. The purified Shp2 biosensor can be phosphorylated by PDGFRβ kinase* in vitro* and undergo subsequent conformational change, causing a corresponding FRET increase (Figure 2A). We utilize this established biochemical reaction between Shp2 biosensor and PDGFRβ kinase to study its reconstitution in protocells, which can be monitor by FRET imaging.

Our preliminary study showed the successful loading of fluorescent Shp2 biosensor into the silica cell replica (Figure 2B). A lipid bilayer membrane can be assembled on the surface of these replicas from liposome fusion (Figure 1A) to create the protocell architecture that mimics both the tortuosity of the internal milieu and also the nanoporous support of the cell membrane. First, silica cell replicas were incubated with the FRET-based Shp2 biosensor and PDGFRβ kinase for protein loading into the pores. Upon the ATP addition, a clear FRET change can be observed in silica cell replicas as ATP can initiate the reaction between the Shp2 biosensor and PDGFRβ kinase (Figure 2A,C), indicating the reconstitution of biochemical reactions inside the porous structure. Notably, there was no lipid bilayer in these silica cell replicas, permitting ATP to diffuse inside freely. In the future, we plan to fuse an ATP-permeable lipid bilayer on the silica cell replicas, incorporating either α-hemolysin or VDAC1, and beyond that a lipid bilayer that will comprehensively mimic the PDGF signaling pathway.

### 3.3. Protocells as Nanocarriers for Targeted Drug Delivery

We have discussed mostly protocells of micrometer size for various* in vitro* reconstitution experiments in the area of basic research. Meanwhile, nanomedicine as a new type of theranostics is revolutionizing our pharmaceutical industry with superior properties compared to conventional free drug treatment [55]. Liposomes as a type of nanocarrier, have been extensively studied and approved for clinical uses [56]. Silica-based protocells of nanometer size have the potential be designed for targeted drug delivery as a new type of nanomedicine [57]. Our group has also pioneered research in this area.

**Figure 3:**
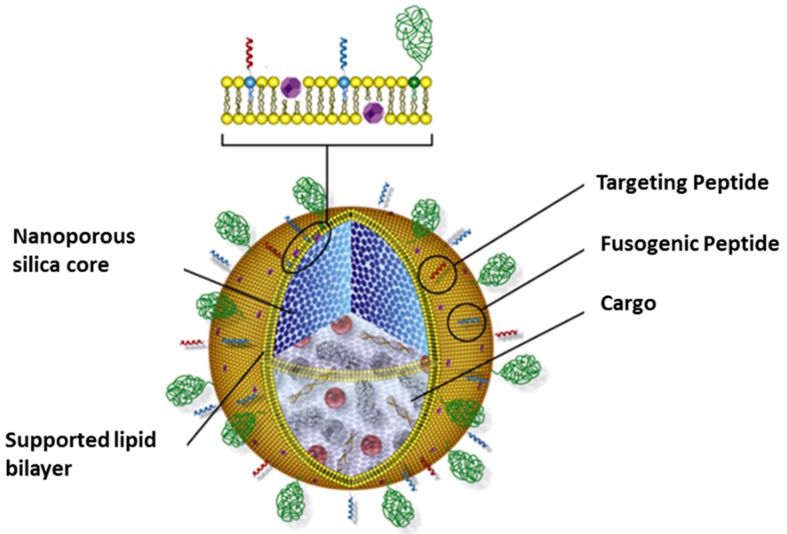
The cartoon depicts the nanoporous silica-based protocell for drug delivery.

We have designed and optimized protocell-based multicomponent cargo delivery, specific to liver cancer cells, combining features of nanoporous silica particles and liposomes [4] (Figure 3). The lipid bilayer was modified with a targeting peptide, a fusogenic peptide, and PEG, to achieve targeting specificity, endosomal escape and extended circulation time, respectively. The nanoporous silica cores (120 nm sphere size, 2.5 nm pore size) were shown to accommodate various therapeutic and diagnostic cargos, such as quantum dots for imaging, chemotherapeutic drugs and nucleic acid. Comparative studies with liposomes of identical bilayer compositions and size were done to demonstrate the advantage of the protocells with respect to features important for nanomedicine: enhanced capacity, selectivity and stability. The improved capacity is a result of the high surface area of the nanoporous silica core while the lipid-silica interaction increases the stability to reduce cargo leakage* in vivo*. In addition, the nanoporous support also increased lateral mobility of molecules such as targeting peptides in the lipid bilayer (as compared to both liposomes or lipid bilayer on non-porous solid support), which contribute to the selectivity and efficiency of the peptide. Together, the synergistic combination of two established and well-studied materials allows high delivery efficiency and improved targeting specificity, which are essential features for effective nanomedicine with maximal target effects and minimal off-target effects.

In particular, protocells were found to bind to hepatocellular carcinoma cells (HCCs) with low targeting-peptide densities while liposomes failed to do so, because the enhanced fluidity of lipid bilayers on the protocell facilitated multivalent peptide binding to the cell surface [58]. This unique advantage of the protocells is critical to enhance specific affinity of the targeting peptide and guide receptor-mediated endocytosis of protocells. We found selective internalization of protocells with low density peptides by HCCs, but not by normal hepatocytes. After endocytosis, the fusogenic peptide on the protocells promotes endosomal escape of protocells and therefore prevents degradation of cargos within the endolysomes. With the successful engineering of the protocells for targeted delivery, Ashley *et al.* delivered high pay loads of various cytotoxic agents to HCCs to test the effectiveness of protocells. For doxorubicin, protocells were reported to have a 1000 times higher capacity (with 90% release) than similar sized liposomes. By contrast, the leakage of liposomes was 90% within 72 h, while protocells could last weeks with less than 20% leakage. In addition, protocells displayed a prolonged release of doxorubicin in endosomal-mimicking buffer. The end results are the effective killing of HCCs by protocells (90%) while more than 90% normal hepatocytes stay alive. This differential effect is only seen with treatment of protocells but not liposomes. Our silica-based protocells show great promise to kill cancer cells* in vitro* and efforts are being made for their* in vivo* testing.

As a new therapeutic agent, small interfering RNA (siRNA) has many advantages over conventional small molecule-based therapy [59]. RNA therapy is potentially exquisitely specific and powerful. Design of siRNA is relatively easy in the genomic age as the sequences of targeted genes and regulatory elements are available. The problem is delivery, because bare RNA is very fragile and cells have enzymes to degrade foreign RNA. Therefore, the key to RNA therapy is protection and targeted delivery. Our group followed up the work on nanoscale protocells and tailored them for siRNA delivery [60]. The key modifications of the protocells for siRNA loading include changing the surface charge of the silica core to positive and increasing the pore size to accommodate siRNA. Through the reaction of silica with an amine-containing silane, the surface of the protocells becomes positive and therefore has strong electrostatic interaction with negatively charged siRNA molecules, which increases the loading capacity more than 10 fold. Using positively charged liposomes for protocell fusion can also enhance the loading of siRNA into unmodified negatively charged silica cores, but this design has higher cytotoxicity than zwitterionic lipids on positive cores. The silica cores (165 nm) in these protocells have a multimodal pore morphology composed of large (23–30 nm), surface-accessible pores interconnected by 3–13 nm ones so that siRNA of 5–6 nm can be loaded. The commercially available cationic lipid nanoparticles were used as the control for comparison. Our modified protocell has higher siRNA loading, enhanced stability as well as slower release, making it a superior nanocarrier for siRNA delivery. With established targeting and fusogenic peptides, siRNAs targeting the cyclin superfamily were successfully delivered to HCCs. They had a dramatic effect, inducing cell growth arrest and apoptosis, confirming the targeted delivery by protocells.

Our vision is that basic research on membrane proteins and cytosol reconstitution in protocells of micrometer size will be translated to protocells of nanometer size, which could potentially extend the range of applications to targeted narrow spectrum antimicrobial therapy.

## 4. Conclusions and Future Prospects

By integrating nanoscience, molecular biosensors and imaging, and biochemistry in the context of systems biology and synthetic biology, we are establishing a protocell platform to reconstitute and model cellular complexity. This will be an important component of a general and systematic approach to understand the design principles of molecular networks in a cellular environment. Protocell-based research will have fundamental implications for understanding information flow in biomolecular networks and directed self-assembly at the nanoscale. The protocell can also serve as a platform for investigating how the properties of membranes depend on their physical environment, and how confined geometries influence biochemical reactions.

The nanoporous silica-based protocell model is part of the foundation for a completely bottom-up synthetic biology, in which all parts of a biomimetic cell can be quantitatively characterized and therefore understood at a level that is impossible in more genomically complete constructs. This work should be regarded as complementary and synergistic to both other synthetic biology work, that begins with a living cell [2,61], or other protocell work, that begins with the concept of a self-replicating chemical network [7,61,62,63]. This is just the beginning of the protocell era. The data generated with the protocells can be readily adapted for computational modeling, which should bridge the gap between* in vitro* and in cell results. Although current progress only demonstrates relatively simple cellular functions, we can readily increase complexity of the system by adding more modules and/or feedback modules. 

Patch clamp experiments on ion channels have not been possible with silica-based protocells as the rigid silica support doesn’t allow the formation of gigaseals for single-channel and whole-cell patch clamp [35]. Conceivably we could construct protocells with a softer core (e.g., organic polymers) or dissolvable core that would help to direct the formation of a cytoskeleton and assemble molecular components and then be removed to enable cell-like mechanical properties. This is a step towards ultimately replacing the rigid silica core with a more biomimetic actin cytoskeleton, which would open the possibilities of motility and replication—beyond the scope of the current functional protocells but indicative of future possibilities. 

The bottom-up cell, especially in the early stages of implementing the approach, is very oversimplified compared to any living cell. Thus current work of the assembly of membrane and intracellular components is only the first step, leading to more complexity as more components are added. Within such protocells we could incorporate the replication machinery to study the origin of life. Ultimately we can incorporate the “genome” into the protocells and start to engineer the supporting structure step by step to replicate any desired function of a living cell. Ultimately the bottom-up and top-down approaches will converge on a minimal living cell, whose operation will be completely understood, because it will have been created *de novo*. Our protocell system has the potential to serve as a significant tool to advance our understanding of the chemistry of life.

## References

[1] Endy D. (2005). Foundations for engineering biology. Nature.

[2] Gibson D.G., Glass J.I., Lartigue C., Noskov V.N., Chuang R.-Y., Algire M.A., Benders G.A., Montague M.G., Ma L., Moodie M.M. (2010). Creation of a bacterial cell controlled by a chemically synthesized genome. Science.

[3] Liu J., Stace-Naughton A., Jiang X., Brinker C.J. (2009). Porous nanoparticle supported lipid bilayers (protocells) as delivery vehicles. J. Am. Chem. Soc..

[4] Ashley C.E., Carnes E.C., Phillips G.K., Padilla D., Durfee P.N., Brown P.A., Hanna T.N., Liu J., Phillips B., Carter M.B. (2011). The targeted delivery of multicomponent cargos to cancer cells by nanoporous particle-supported lipid bilayers. Nat. Mater..

[5] Oberholzer T., Luisi P.L. (2002). The use of liposomes for constructing cell models. J. Biol. Phys..

[6] Adamala K., Luisi P.L. (2012). Experimental systems to explore life origin: perspectives for understanding primitive mechanisms of cell division. Results Probl. Cell Differ..

[7] Szostak J.W., Bartel D.P., Luisi P.L. (2001). Synthesizing life. Nature.

[8] Rigaud J.L., Levy D. (2003). Reconstitution of membrane proteins into liposomes. Methods Enzymol..

[9] Oberholzer T., Albrizio M., Luisi P.L. (1995). Polymerase chain reaction in liposomes. Chem. Biol..

[10] Kumar R.K., Yu X., Patil A.J., Li M., Mann S. (2011). Cytoskeletal-like Supramolecular Assembly and Nanoparticle-Based Motors in a Model Protocell. Angew. Chem. Int. Ed..

[11] Stano P., Carrara P., Kuruma Y., Pereira de Souza T., Luisi P.L. (2011). Compartmentalized reactions as a case of soft-matter biotechnology: Synthesis of proteins and nucleic acids inside lipid vesicles. J. Mater. Chem..

[12] Kita H., Matsuura T., Sunami T., Hosoda K., Ichihashi N., Tsukada K., Urabe I., Yomo T. (2008). Replication of Genetic Information with Self-Encoded Replicase in Liposomes. Chembiochem.

[13] Hammer D.A., Kamat N.P. (2013). Towards an artificial cell. FEBS Lett..

[14] Huang X., Li M., Green D.C., Williams D.S., Patil A.J., Mann S. (2013). Interfacial assembly of protein-polymer nano-conjugates into stimulus-responsive biomimetic protocells. Nat. Commun..

[15] Huang X., Patil A.J., Li M., Mann S. (2014). Design and Construction of Higher-Order Structure and Function in Proteinosome-Based Protocells. J. Am. Chem. Soc..

[16] Martino C., Kim S.H., Horsfall L., Abbaspourrad A., Rosser S.J., Cooper J., Weitz D.A. (2012). Protein expression, aggregation, and triggered release from polymersomes as artificial cell-like structures. Angew. Chem. Int. Ed..

[17] Li M., Huang X., Tang T.-Y.D., Mann S. (2014). Synthetic cellularity based on non-lipid micro-compartments and protocell models. Curr. Opin. Chem. Biol..

[18] Kumar R.K., Li M., Olof S.N., Patil A.J., Mann S. (2013). Artificial Cytoskeletal Structures Within Enzymatically Active Bio-inorganic Protocells. Small.

[19] Li M., Harbron R.L., Weaver J.V.M., Binks B.P., Mann S. (2013). Electrostatically gated membrane permeability in inorganic protocells. Nat. Chem..

[20] Markstrom M., Gunnarsson A., Orwar O., Jesorka A. (2007). Dynamic microcompartmentalization of giant unilamellar vesicles by sol-gel transition and temperature induced shrinking/swelling of poly(N-isopropyl acrylamide). Soft Matter..

[21] Tang T.-Y.D., Hak C.R.C., Thompson A.J., Kuimova M.K., Williams D.S., Perriman A.W., Mann S. (2014). Fatty acid membrane assembly on coacervate microdroplets as a step towards a hybrid protocell model. Nat. Chem..

[22] Kaehr B., Townson J.L., Kalinich R.M., Awad Y.H., Swartzentruber B.S., Dunphy D.R., Brinker C.J. (2012). Cellular complexity captured in durable silica biocomposites. Proc. Natl. Acad. Sci. USA.

[23] Pontani L.L., van der Gucht J., Salbreux G., Heuvingh J., Joanny J.F., Sykes C. (2009). Reconstitution of an actin cortex inside a liposome. Biophys. J..

[24] Takiguchi K., Yamada A., Negishi M., Tanaka-Takiguchi Y., Yoshikawa K. (2008). Entrapping desired amounts of actin filaments and molecular motor proteins in giant liposomes. Langmuir.

[25] Nomura S.M., Tsumoto K., Hamada T., Akiyoshi K., Nakatani Y., Yoshikawa K. (2003). Gene expression within cell-sized lipid vesicles. Chembiochem.

[26] Stachowiak J.C., Richmond D.L., Li T.H., Li A.P., Parekh S.H., Fletcher D.A. (2008). Unilamellar vesicle formation and encapsulation by microfluidic jetting. Proc. Natl. Acad. Sci. USA.

[27] Ellis R.J. (2001). Macromolecular crowding: Obvious but underappreciated. Trends Biochem. Sci..

[28] Ellis R.J., Minton A.P. (2003). Cell biology: Join the crowd. Nature.

[29] Zhou H.X., Rivas G., Minton A.P. (2008). Macromolecular crowding and confinement: Biochemical, biophysical, and potential physiological consequences. Annu. Rev. Biophys..

[30] Bhatia R.B., Brinker C.J. (2000). Aqueous sol-gel process for protein encapsulation. Chem. Mater..

[31] Brinker C.J. (1988). Hydrolysis and Condensation of Silicates—Effects on Structure. J. Non-Cryst. Solids.

[32] Natarajan S.K., Selvaraj S. (2014). Mesoporous silica nanoparticles: Importance of surface modifications and its role in drug delivery. RSC Adv..

[33] Lu Y., Fan H., Stump A., Ward T.L., Rieker T., Brinker C.J. (1999). Aerosol-assisted self-assembly of mesostructured spherical nanoparticles. Nature.

[34] Brinker C.J., Lu Y., Sellinger A., Fan H. (1999). Evaporation-induced self-assembly: Nanostructures made easy. Adv. Mater..

[35] Davis R.W., Flores A., Barrick T.A., Cox J.M., Brozik S.M., Lopez G.P., Brozik J.A. (2007). Nanoporous microbead supported bilayers: Stability, physical characterization, and incorporation of functional transmembrane proteins. Langmuir.

[36] Sackmann E. (1996). Supported membranes: Scientific and practical applications. Science.

[37] Troutier A.L., Ladaviere C. (2007). An overview of lipid membrane supported by colloidal particles. Adv. Colloid Interface Sci..

[38] Koenig B.W., Krueger S., Orts W.J., Majkrzak C.F., Berk N.F., Silverton J.V., Gawrisch K. (1996). Neutron Reflectivity and Atomic Force Microscopy Studies of a Lipid Bilayer in Water Adsorbed to the Surface of a Silicon Single Crystal. Langmuir.

[39] Johnson S.J., Bayerl T.M., Weihan W., Noack H., Penfold J., Thomas R.K., Kanellas D., Rennie A.R., Sackmann E. (1991). Coupling of spectrin and polylysine to phospholipid monolayers studied by specular reflection of neutrons. Biophys. J..

[40] Varma S., Teng M., Scott H.L. (2012). Nonintercalating nanosubstrates create asymmetry between bilayer leaflets. Langmuir.

[41] Nordlund G., Ng J.B.S., Bergström L., Brzezinski P. (2009). A membrane-reconstituted multisubunit functional proton pump on mesoporous silica particles. ACS Nano.

[42] Oliynyk V., Mille C., Ng J.B.S., von Ballmoos C., Corkery R.W., Bergström L. (2013). Selective and ATP-driven transport of ions across supported membranes into nanoporous carriers using gramicidin A and ATP synthase. Phys. Chem. Chem. Phys..

[43] Stano P., Pereira de Souza T., Carrara P., Altamura E., D’Aguanno E., Caputo M., Luisi P.L., Mavelli F. (2014). Recent Biophysical Issues about the Preparation of Solute-Filled Lipid Vesicles. Mech. Adv. Mater. Struct..

[44] Noireaux V., Libchaber A. (2004). A vesicle bioreactor as a step toward an artificial cell assembly. Proc. Natl. Acad. Sci. USA.

[45] Rostovtseva T., Colombini M. (1997). VDAC channels mediate and gate the flow of ATP: Implications for the regulation of mitochondrial function. Biophys. J..

[46] Hiller S., Garces R.G., Malia T.J., Orekhov V.Y., Colombini M., Wagner G. (2008). Solution structure of the integral human membrane protein VDAC-1 in detergent micelles. Science.

[47] Yu T.Y., Raschle T., Hiller S., Wagner G. (2012). Solution NMR spectroscopic characterization of human VDAC-2 in detergent micelles and lipid bilayer nanodiscs. Biochim. Biophys. Acta.

[48] Kuruma Y., Stano P., Ueda T., Luisi P.L. (2009). A synthetic biology approach to the construction of membrane proteins in semi-synthetic minimal cells. Biochim. Biophys. Acta.

[49] Hartmann M. (2005). Ordered Mesoporous Materials for Bioadsorption and Biocatalysis. Chem. Mater..

[50] Tallquist M., Kazlauskas A. (2004). PDGF signaling in cells and mice. Cytokine Growth Factor Rev..

[51] Chan G., Kalaitzidis D., Neel B.G. (2008). The tyrosine phosphatase Shp2 (PTPN11) in cancer. Cancer Metastasis Rev..

[52] Loh M.L., Vattikuti S., Schubbert S., Reynolds M.G., Carlson E., Lieuw K.H., Cheng J.W., Lee C.M., Stokoe D., Bonifas J.M. (2004). Mutations in PTPN11 implicate the SHP-2 phosphatase in leukemogenesis. Blood.

[53] Tartaglia M., Gelb B.D. (2005). Noonan syndrome and related disorders: Genetics and pathogenesis. Annu. Rev. Genomics Hum. Genet..

[54] Sun J., Lu S., Ouyang M., Lin L.J., Zhuo Y., Liu B., Chien S., Neel B.G., Wang Y. (2013). Antagonism between binding site affinity and conformational dynamics tunes alternative cis-interactions within Shp2. Nat. Commun..

[55] Kim B.Y., Rutka J.T., Chan W.C. (2010). Nanomedicine. New Engl. J. Med..

[56] Slingerland M., Guchelaar H.J., Gelderblom H. (2011). Liposomal drug formulations in cancer therapy: 15 years along the road. Drug Discov. Today.

[57] Yang Y., Song W., Wang A., Zhu P., Fei J., Li J. (2010). Lipid coated mesoporous silica nanoparticles as photosensitive drug carriers. Phys. Chem. Chem. Phys..

[58] Kendall E.L., Ngassam V.N., Gilmore S.F., Brinker C.F., Parikh A.N. (2013). Lithographically defined macroscale modulation of lateral fluidity and phase separation realized via patterned nanoporous silica-supported phospholipid bilayers. J. Am. Chem. Soc..

[59] Miele E., Spinelli G.P., Miele E., di Fabrizio E., Ferretti E., Tomao S., Gulino A. (2012). Nanoparticle-based delivery of small interfering RNA: Challenges for cancer therapy. Int. J. Nanomed..

[60] Ashley C.E., Carnes E.C., Epler K.E., Padilla D.P., Phillips G.K., Castillo R.E., Wilkinson D.C., Wilkinson B.S., Burgard C.A., Kalinich R.M. (2012). Delivery of small interfering RNA by peptide-targeted mesoporous silica nanoparticle-supported lipid bilayers. ACS Nano.

[61] Luisi P.L. (2002). Toward the engineering of minimal living cells. Anat. Rec..

[62] Luisi P.L., Ferri F., Stano P. (2006). Approaches to semi-synthetic minimal cells: A review. Naturwissenschaften.

[63] Ichihashi N., Matsuura T., Kita H., Sunami T., Suzuki H., Yomo T. (2010). Constructing Partial Models of Cells. Cold Spring Harb. Persp. Biol..

